# Journal of Cardiovascular Magnetic Resonance: 2017/2018 in review

**DOI:** 10.1186/s12968-019-0594-8

**Published:** 2019-12-30

**Authors:** Warren J. Manning

**Affiliations:** Beth Israel Deaconess Medical Center, Harvard Medical School, Boston, Massachusetts USA

**Keywords:** Cardiovascular magnetic resonance, Review, Editorial process, Imaging

## Abstract

There were 89 articles published in the *Journal of Cardiovascular Magnetic Resonance* (*JCMR*) in 2017, including 76 original research papers, 4 reviews, 5 technical notes, 1 guideline, and 3 corrections. The volume was down slightly from 2017 with a corresponding 15% decrease in manuscript submissions from 405 to 346 and thus reflects a slight increase in the acceptance rate from 25 to 26%. The decrease in submissions for the year followed the initiation of the increased author processing charge (APC) for Society for Cardiovascular Magnetic Resonance (SCMR) members for manuscripts submitted after June 30, 2018. The quality of the submissions continues to be high. The 2018 JCMR Impact Factor (which is published in June 2019) was slightly lower at 5.1 (vs. 5.46 for 2017; as published in June 2018. The 2018 impact factor means that on average, each *JCMR* published in 2016 and 2017 was cited 5.1 times in 2018. Our 5 year impact factor was 5.82.

In accordance with Open-Access publishing guidelines of BMC, the *JCMR* articles are published on-line in a continuus fashion in the chronologic order of acceptance, with no collating of the articles into sections or special thematic issues. For this reason, over the years, the Editors have felt that it is useful for the *JCMR* audience to annually summarize the publications into broad areas of interest or themes, so that readers can view areas of interest in a single article in relation to each other and contemporaneous *JCMR* publications. In this publication, the manuscripts are presented in broad themes and set in context with related literature and previously published *JCMR* papers to guide continuity of thought within the journal. In addition, as in the past two years, I have used this publication to also convey information regarding the editorial process and as a “State of our *JCMR*.”

This is the 12th year of *JCMR* as an open-access publication with BMC (formerly known as Biomed Central). The timing of the *JCMR* transition to the open access platform was “ahead of the curve” and a tribute to the vision of Dr. Matthias Friedrich, the SCMR Publications Committee Chair and Dr. Dudley Pennell, the *JCMR* editor-in-chief at the time. The open-access system has dramatically increased the reading and citation of *JCMR* publications and I hope that you, our authors, will continue to send your very best, high quality manuscripts to *JCMR* for consideration. It takes a village to run a journal and I thank our very dedicated Associate Editors, Guest Editors, Reviewers for their efforts to ensure that the review process occurs in a timely and responsible manner. These efforts have allowed the *JCMR* to continue as the premier journal of our field. This entire process would also not be possible without the dedication and efforts of our managing editor, Diana Gethers. Finally, I thank you for entrusting me with the editorship of the *JCMR* as I begin my 4th year as your editor-in-chief. It has been a tremendous experience for me and the opportunity to review manuscripts that reflect the best in our field remains a great joy and highlight of my week!

The *Journal of Cardiovascular Magnetic Resonance* (*JCMR*) is a publication of the Society for Cardiovascular Magnetic Resonance (SCMR). There were 89 articles published in JCMR in 2018, including 76 original research papers, 4 reviews, 5 technical notes, 1 guideline, and 3 corrections. The volume was down slightly from 2017 with a corresponding 15% decrease in manuscript submissions from 405 to 346. As a result, there was a slight increase in the acceptance rate from 25 to 26%. The decrease in submissions for the 2018 year followed the initiation of an author processing charge (APC) for SCMR members for manuscripts submitted after June 30, 2018. The $500/manuscript charge for SCMR members reflects an 80% discount to the full APC of $2500. Our largest source of submissions continues to be the United States, followed by China, the United Kingdom and Germany (Fig. 1). The top four publication countries were the United States, United Kingdom, China, and Germany (Fig. 2).

**Fig. 1:**
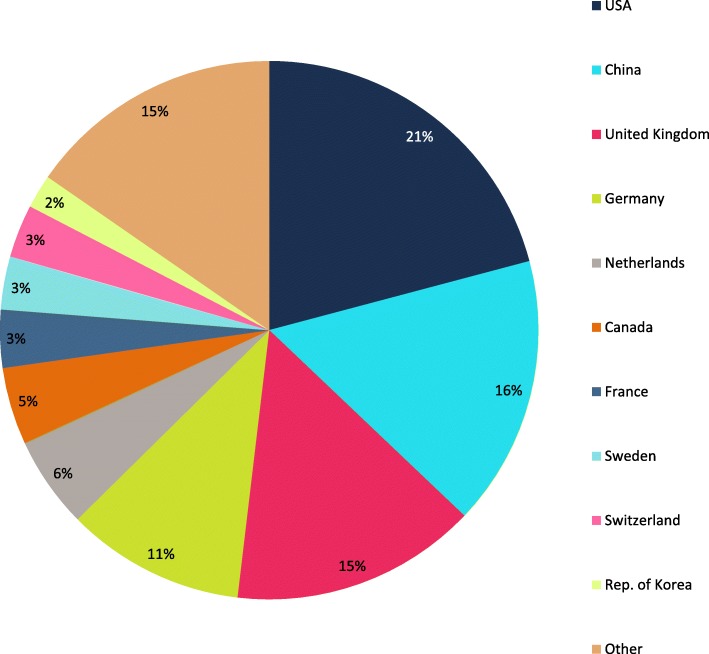
Source of *JCMR* 2018 manuscript submissions by country

**Fig. 2:**
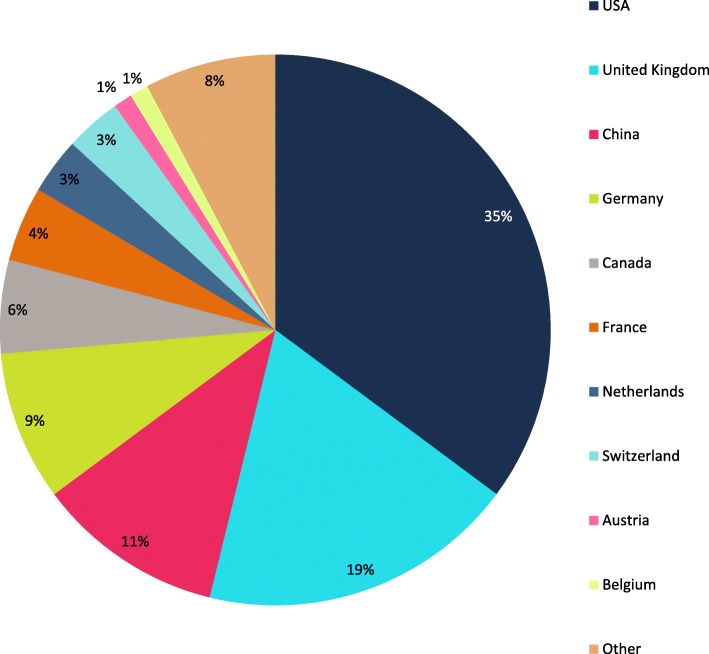
Source of *JCMR* 2018 manuscript acceptances by country

The quality of *JCMR* submissions continues to be high as reflected by our Impact Factor. The 2018 *JCMR* Impact Factor (which is published in June 2019) was slightly lower at 5.1 (vs. 5.46 for 2017), which is the third highest impact factor ever recorded for *JCMR*. The 2018 impact factor means that the *JCMR* papers that were published in 2016 and 2017 were cited on average 5.1 times in 2018. This puts *JCMR* well positioned in the top quartile of journals in the broad categories of “Cardiac and Cardiovascular systems” and “Radiology, Nuclear medicine and Medical Imaging.” Most importantly, the open-access format allows for much greater visibility for our authors with *JCMR* digital accesses now exceeding 1,000,000 per year – an threshold simply not achievable with our prior subscription print publication.

## *JCMR* editorial office/manuscript handling

Since December 2016, the *JCMR* editorial office has been located at the Beth Israel Deaconess Medical Center, Boston, Massachusetts, USA under the leadership of its third editor-in-chief, Dr. Warren J Manning. Dr. Gerald Pohost was the *JCMR* inaugural editor-in-chief. In 2006, he was succeeded *by* Professor Dudley Pennell of the Royal Brompton Hospital, London, England.

The current *JCMR* associate editors reflect the international and diverse spectrum of the SCMR. This past year, Dr. Andrew Powell stepped down from his associate editor *JCMR* position to undertake the SCMR presidency. His position on the associate editorial board was filled by Dr. Joshua Robinson, Northwestern University, Chicago, Illinois, USA. The other associate editors include Drs. Rene Botnar (UK/Chile), John Greenwood (UK), Yuchi Han (USA), Dara Kraichman (USA), Robert Lederman (USA), Timothy Leiner (The Netherlands), and Reza Nezafat (USA). In addition, Dr. Long Ngo (USA) serves as the *JCMR* statistical editor. Drs. Amit Patel (USA) and Juan Lopez-Mattei (USA) serve as our social media/Twitter editors. Dr. Timothy Leiner will be stepping down from his associate editor role in mid-2020 as he undertakes the presidency of the International Society of Magnetic Resonance in Medicine (ISMRM). We thank Tim for his efforts these past 3 years and wish him continued success with the ISMRM presidency. Diana Gethers (jcmroffice@scmr.org) continues to serve as our managing editor. All correspondence to the *JCMR* managing office should continue to be sent to jcmroffice@scmr.org.

## Manuscript review process

All manuscripts are submitted and processed through the www.jcmr-online.org website.

After initial BMC office confirmation that the manuscript is in the appropriate format (abstract, text, references, figures, tables), the manuscript is sent for initial review to the Boston office. Within 48 h, I assess the manuscript for its appropriateness for the *JCMR* readership and a determination as to its overall likely priority for publication. Approximately 5-10% of submitted manuscripts are deemed inappropriate or very unlikely to reach sufficient priority for acceptance and are therefore returned to the author(s) within a week so as to expedite submission to a more appropriate journal. If appropriate, the authors are also offered the opportunity to directly forward their manuscript to another BMC open access publication.

For manuscripts deemed appropriate for consideration, an associate editor is then assigned and reviewer assignments are then requested. Manuscript evaluations are simultaneously requested from multiple reviewers until 3 reviewers have confirmed acceptance. Reviewers are asked to follow a specific format [see below] and to return their review within 2 weeks of acceptance. We are fortunate to have nearly 1400 registered reviewers (but are continuously interested in expanding our reviewer pool and encourage all members/innovators/leaders of the CMR field to apply to be a reviewer. If you are interested in becoming a *JCMR* reviewer, please contact our managing editor, Diana Gethers [jcmroffice@scmr.org] or visit us at the *JCMR* display at the 2020 SCMR annual scientific meeting in Orlando*.* Conflict manuscripts, those for which a member of the associate editorial board is either an author or closely associated with an author, are independently handled by a Guest Editor (Table 1). If the manuscript is accepted, the Guest Editor is recognized in the publication.

**Table 1:** 2018 *JCMR* Guest Editors

Hakan Arheden	
Albert de Roos	
Francesca Nesta Delling	
Mark Fogel	
James Hamilton	
Thomas H Hauser	
Sebastian Kelle	
Peter Kellman	
Christopher M. Kramer	
Hildo Lamb	
Eike Nagel	
Stefan Neubauer	
Dana Peters	
Martin Prince	
Nathaniel Reichek	
John Ridgway	
Michael Salerno	
Juerg Schwitter	
Matthias Stuber	
Connie Tsao	
Anne Valente	
Robert Weiss	
Graham Wright	

When at least two (of 3 agreed) reviews have been received by noon Friday, a manuscript is scheduled to be discussed at our associate editorial board meeting held every Tuesday from 9:30-10:30 am EST. When I am out of town/unavailable, the associate editors continue to meet at that time so as to not delay the publication process. At each meeting, 4-12 manuscripts may be discussed. The manuscript decisions at that meeting include

1. Accept
2. Minor revision – no new experiments are needed, relatively minor text changes or analyses are requested; 30 day turn-around; > 98% acceptance is anticipated
3. Major revision – substantial text and/or analyses needed, a few additional experiments; 90 day turn-around; ~ 60% acceptance is anticipated
4. Denovo resubmission – substantial new experiments/analyses are needed; unlimited turn-around; ~ 40% acceptance is anticipated
5. Decline – authors are offered the opportunity to have their manuscript considered by another journal in the BMC family with inclusion of the *JCMR* reviews to expedite the process.

When a manuscript is accepted, I then edit the submission for *JCMR* style/abbreviations before final submission to BMC for typesetting. The galleys are then sent to the corresponding author and then to me for final sign-off. I then identify a fingernail image for publication in *JCMR* to accompany the tweet. The manuscript is usually published on-line within a week of my final sign-off.

Our target goal is than 60% of manuscripts will have a submission to first decision within 31 days, a process that is very dependent on timely reviews. If the two reviews markedly differ in their assessment/recommendation (~ 25% of the time) or the associate editor feels we need additional information, we may delay a decision until the third review has been received or solicit a fourth reviewer – a process that unfortunately can add a month or more to the review process. At our editorial meeting, we may also to seek the counsel of our statistical reviewer. We try to alert the corresponding author if this situation occurs or the unusual occurance of our not being able to discuss all of the manuscripts on our weekly agenda (or the assigned associate editor is unable to participate).

As with any review process, we recognize that we are not perfect. We do our best to objectively assess the science, presentation, and appropriateness for *JCMR*. Is the study scientifically sound? Does the manuscript present new data? Will our readership be interested or informed by the topic?

Reviews are currently anonymous and not available to our readers, but we are exploring the ability to include *anonymized* reviews with published manuscripts. I do not anticipate publication of submitted (but not accepted manuscripts) or inclusion of prior versions of an accepted manuscript with reviews as I am concerned this may be confusing to the reader.

## Reviewer instructions – what makes a good review?

Like all peer review journals, the *JCMR* is dependent on reviewers to provide an independent evaluation of the quality (innovation, study design, data analysis, presentation) of a submission. Though our associate editor knowledge base is broad, they are not experts in all areas. We expect reviewers to act independently and to acknowledge any conflicts of interest.

What makes a good review? I find an article by my friend and colleague, Dr. Anthony DeMaria, former editor-in-chief of the *Journal of the American College of Cardiology* to be a very valuable resource (http://www.onlinejacc.org/content/42/7/1314). The *JCMR* review invitation includes the broad strokes of what makes a good review. This includes

1. Synopsys of the manuscript’s overall **significance to the field**
2. **Novelty/originality** of the work
3. Is the **study design** appropriate
4. Soundness of the **Methods**
5. Are the **statistical methods** appropriate
6. Are the **Results** presented in a logical and succinct manner
7. Are the **Figures** and T**ables** appropriate. Is there redundancy in the text and Figures/Tables. If so, how are the data most appropriately presented for the reader?
8. Are the **Discussion** and **Limitations** section appropriate both in length and content
9. Listing of **References** that may be overlooked or misquoted
10. **Ease of reading** – is editing by native English speaker needed

Each primary manuscript review (i.e., not revisions) that we receive is subjectively assessed by the associate editor based on the criteria above. A grade of ≥60% would qualify the reviewer for the Gold Star Reviewer award (see next). Reviewers with consistent grades of < 50% are no longer asked to be reviewers.

## Reviewer recognition – Gold Star reviewers

**Fig. 3:**
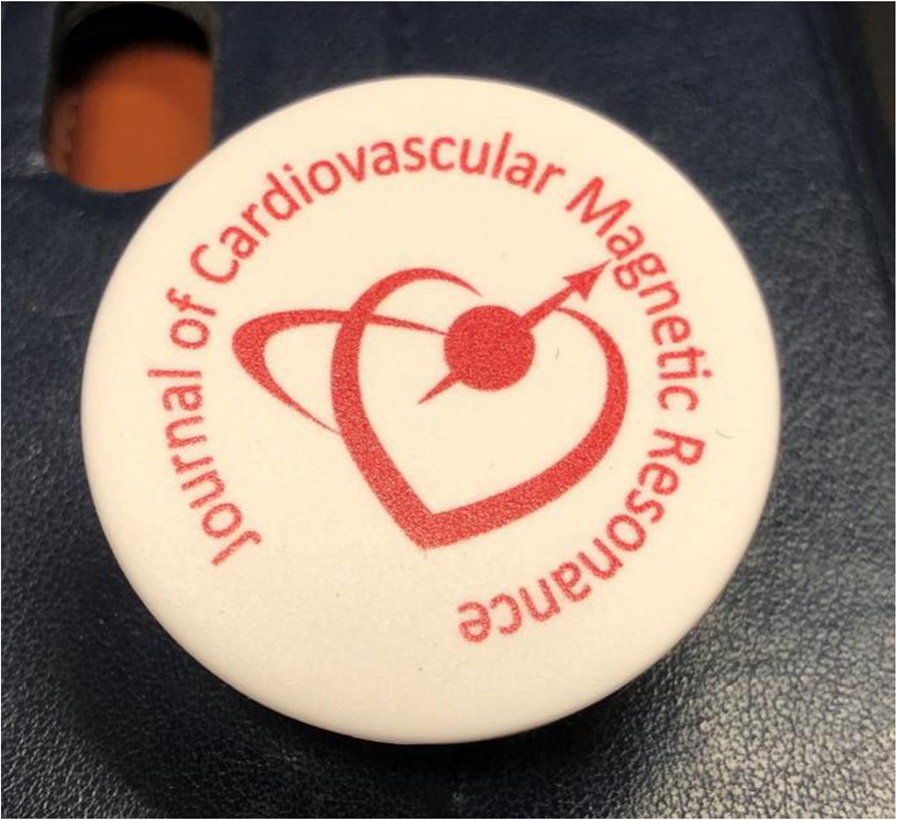
In 2018, JCMR Gold Star Reviewers were offered a phone holder pop-up with the *JCMR logo*

Reviewers are a key component to the success of the *JCMR*. As a recognition of reviewers, at the 2019 SCMR Annual meeting in Beelevue, Washington, USA we recognized our 94 “Gold Star” Reviewers for 2018 (Table 2). Gold Star reviewers are those individuals who reviewed at least 3 *JCMR* manuscripts in 2018, submitted their review on-time, and submitted a high quality review as defined above. In addition to public recognition at the meeting (Gold Star ribbon, *JCMR* booth listing, and intermission slide listing), each Gold Star Reviewer was offered a small gift (Fig. 3) as a token of our appreciation. We plan to continue the Gold Star Reviewer recognition at the upcoming 2020 SCMR Annual meeting in Orlando. Please join the ranks of *JCMR* reviewers and strive to be a Gold Star reviewer! As an added incentive, reviewer continuing medical education (CME) credit started in 2019.

**Table 2:** 2018 *JCMR* Gold Medal Reviewers

Per M Arvidsson	
Patricia Bandettini	
Jessica A. M. Bastiaansen	
Nicoleta Baxan	
Giovanni Biglino	
Kenneth Bilchick	
Dominik Buckert	
Marcus Carlsson	
YuCheng Chen	
Michael Chuang	
Henry Chubb	
Taylor Chung	
Jeremy D Collins	
Christakis Constantinides	
Francisco Contijoch	
Erica Dall’Armellina	
Michael Samuel Dodd	
Chong Duan	
Michael Elliott	
Emil Knut Stenersen Espe	
Ahmed Fahmy	
Paul Finn	
Christopher J Francois	
Julio Garcia	
Nilesh R Ghugre	
James W Goldfarb	
Christopher Haggerty	
Ahmed Hamimi	
Markus Henningsson	
Lazaro Eduardo Hernandez	
Bobak Heydari	
Alexander Hirsch	
Peng Hu	
Edward Hulten	
Tevfik F Ismail	
Jihye Jang	
Michael Jerosch-Herold	
Ning Jin	
Jason Nathaniel Johnson	
Dinesh Kalra	
Keigo Kawaji	
Peter Kellman	
Won Yong Kim	
Anja van der Kolk	
Grigorios Korosoglou	
Sebastian Kozerke	
Rebecca Kozor	
Ramkumar Krishnamurty	
Kasper Kyhl	
Seung-Pyo Lee	
Christine Lorenz	
Jimmy Lu	
Minjie Lu	
Pierre-Yves Marie	
Michael Markl	
Daniel R Messroghli	
Jack Miller	
Mehdi Hedjazi Moghari	
James C Moon	
Francois-Pierre Mongeon	
Vivek Muthurangu	
AV Naumova	
Christopher Nguyen	
Declan O’Regan	
John Oshinski	
Bernard Paelinck	
Dana Peters	
Amir Ali Rahsepar	
Philip Robson	
Giles Roditi	
Tobias Rutz	
Hajime Sakuma	
Michael Schär	
Timothy Slesnick	
Sahar Soleimanifard	
Pascal Spincemaille	
Eric G. Stinson	
Jordan B. Strom	
Avan Suinesiaputra	
Peter P Swoboda	
Connie Tsao	
Ruud B van Heeswijk	
Moriel Vandsburger	
Miguel Silva Vieira	
Adriana Villa	
Gustavo Jardim Volpe	
Sebastian Weingärtner	
Jos J Westenberg	
Jelmer Wolterink	
Timothy C Wong	
Katherine Wu	
Yibin Xie	
Jérôme Yerly	
Alistair Young

## Continuing medical education (CME) and upcoming *JCMR* Journal Club

In late 2017, we introduced on-line CME credit for the benefit of our clinician readers. This program has been a great success and now includes 17 manuscripts (http://scmr.peachnewmedia.com/store/provider/custompage.php?pageid=20). As mentioned, in 2019, we started offering CME credit as a benefit for our reviewers. New for 2020 is a monthly *JCMR* Journal Club moderated by Drs. Scott Flamm (clinical radiology), Raymond Kwong (clinical cardiology) and Matthias Stuber (non-clinical). Journal Club will be on the 2nd Wednesday of the month from 10 to 11 am EST, beginning Wednesday, January 8th. Stay tuned for this exciting new *JCMR* feature. We are investigating CME credit for this offering as well.

### Editorial board

*JCMR e*ditorial board members are leaders in the CMR field and are expected to review up to 4 manuscripts/year. In 2019, we added 6 new members to the Editorial Board - Drs. Frederick Epstein (University of Virginia, USA), Paul Finn (University of California at Los Angeles, USA), Peng Hu (University of California at Los Angeles, USA), Saul Myerson (Oxford University, UK), Matthias Stuber (University of Lausanne, Switzerland) and Claudia Prieto Vasquez (United Kingdom-Chile). For the past year, the *JCMR* Editorial Board has consisted of 45 members with expertise across the spectrum and geography of CMR.

### Social media

The *JCMR* continues to be active on Twitter with the handle “**JournalofCMR**”. This relationship is coordinated by Drs. Amit Patel and Juan Lopez-Mattei. As of late November 2019, we had 2434 followers (a 34% increase over last year). For comparison, the *Journal of the American Society of Echocardiography* (*JASE*) has 842 followers, the *Journal of Cardiac Computed Tomography* (*JCCT*) has 1608 followers, the *Journal of Nuclear Cardiology* has 496 followers, and *Circulation CV Imaging* has 1185 followers. To date this year, we have had 267,877 impressions (a 28% increase from 2018). I applaud the efforts of our social media editors, Drs. Amit Patel and Juan Lopez-Mattei for their efforts in this arena!

### Article processing charge (APC)

The biggest change for *JCMR* authors in 2018 was the APC. Though SCMR members do not receive favorable treatment with regards to manuscript evaluation in the review process, for the first 10 years of our open access publication, the SCMR covered the entire APC for SCMR members. With the ongoing growth of the *JCMR* and SCMR, it became apparent that this benefit was creating an increasing SCMR financial burden that benefited a very small minority of the membership. In addition, there is increasing recognition and expectation by granting agencies that original manuscripts will be published in an open access format for which APC charges are a recognized expense. As a result, the *JCMR* APC policy changed for all manuscripts submitted after June 30, 2018 to include a $500 APC charge for manuscripts submitted by SCMR members. This represents a substantial (80%) discount to the full APC of $2500/manuscript for non-members.

## Case reports

The *JCMR* does not publish individual case reports. Those interested in publishing a CMR case report are encouraged to submit their work to the case series on the SCMR web site (https://scmr.org/page/caseoftheweekLDGPG). In collaboration with the SCMR case of the week editors, we hope to publish a compendium of the 2019 SCMR case reports in 2020 so as to provide a searchable database for those interested.

## Reviews

The *JCMR* does accept unsolicited reviews. For those interested in writing a review, please check with the editor before embarking on this endeavor.

### Pohost and Pennell awards

In recognition of the efforts of our inaugural editor-in-chief, Dr. Gerald M. Pohost, for the past 12 years, the *JCMR* has awarded the Pohost Prize to that manuscript deemed by the associate editors and editorial board to be the best manuscript published in the prior year. At the 2019 annual meeting in Bellevue, Washington, the 12th Gerald M. Pohost Award was presented to Wenjia Bai and colleagues for the manuscript, “Automated cardiovascular magnetic resonance image analysis with fully convolutional networks.” [1]. We also awarded a Pohost “runner up” award to Dr. Rui Guo and co-workers for their manuscript, “Three-dimensional free breathing whole heart cardiovascular magnetic resonance T1 mapping at 3T.” [2]. At that meeting, we presented the 2nd Pennell Award in recognition of our 2nd Editor-in-Chief, Professor Dudley J. Pennell's focus and success on improving the *JCMR* impact factor. The Pennell award is for that *original manuscript* that has most contributed to the *Journal’s* impact factor for the calendar year 3 years prior to the award. The 2nd Dudley J. Pennell Award was presented to Dr. Florian Andre et al. for the manuscript, “Age and gender-related normal left ventricular deformation assessed by cardiovascular magnetic resonance feature tracking.” [3] with a runner-up award to Dr. Oliver Bruder and colleagues for their manuscript “2015 update on acute adverse reactions to gadolinium based contrast agents in cardiovascular MR. Large multi-national and multi-ethnical population experience with 37788 patients from the EurorCMR Registry.” [4] Stay tuned for the 13th Pohost and 3rd Pennell Awards that will presented at the 22nd Scientific Sessions of the *Society* in Orlando this February!

### 2017 And 2018 *JCMR* publications

**Fig. 4:**
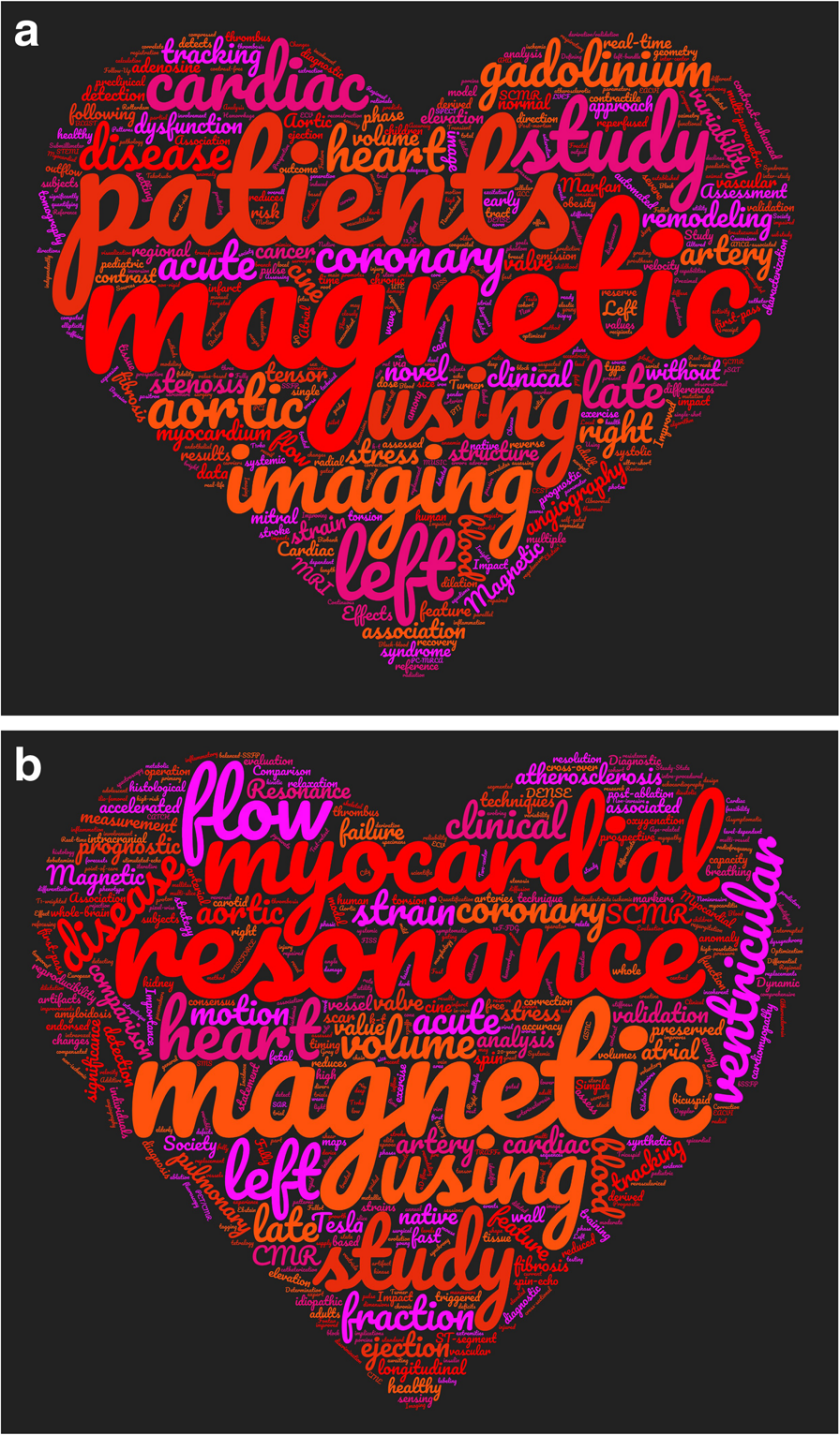
Wordplot derived from the titles of the **a**) 2017 and **b**) 2018 JCMR publications

Over the years, the Editors have felt that it is useful for the *JCMR* audience to annually summarize the publications [5, 6] in broad areas of interest or themes, so that readers can view areas of interest in a single article in relation to each other and contemporaneous *JCMR* publications. A wordplot based on the 2017 and 2018 *JCMR* titles (Fig. 4) suggests an increase in the title words “flow,” “perfusion,” and “mapping” for 2018. Listed below are the 2017 and 2018 *JCMR* publications presented in thematic format with the references for 2017 publications and brief manuscript synopsis for 2018 publications.

## Cardiomyopathies/heart failure

Elucidation of etiology and prognosis in cardiomyopathies continues to be a primary clinical indication for CMR. For 2017, there were publications involving the role of CMR in post-partem cardiomyopathy [7], post-transplant [8], vasculidities [9], arrhythmogenic right ventricular cardiomyopathy [10], cardiac amyloidosis [11], hypertrophic cardiomyopathy [12], and cardio-oncology [13–16]. Publications in 2018 included:

**Association between myocardial extracellular volume and strain analysis through cardiovascular magnetic resonance with histological myocardial fibrosis in patients awaiting heart transplantation**.

In this small study of 12 patients with a dilated cardiomyopathy (DCM) and 10 with ischemic cardiomyopathy undergoing heart transplantation, Cui and co-workers [17] found a strong relationship between extracellular volume fraction (ECV) and histological collagen volume fraction. Native T2, global longitudinal strain, global circumferential strain, and global radial strain were not associated with ECV or collagen volume fraction.

**Myocardial tissue characterization and strain analysis in healthy pregnant women using cardiovascular magnetic resonance native T1 mapping and feature tracking technique**.

In this prospective study, Nii and colleagues [18] performed serial CMR with native T1 mapping in 12 normal pregnant woman during the 2nd and 3rd trimesters and 1 month post-partum. They found left ventricular (LV) remodeling during normal pregnancy was associated with an increase in LV mass without edema or fibrosis or contractile dysfunction.

**Associations and prognostic significance of diffuse myocardial fibrosis by cardiovascular magnetic resonance in heart failure with preserved ejection fraction**.

Heart failure with preserved ejection fraction (HFpEF) remains a diagnostic and therapeutic challenge with increasing focus on imaging for guidance. Roy and co-workers [19] prospectively performed CMR in 118 subjects with HFpEF and found ECV to be increased in the HFpEF cohort. The addition of ECV to diabetes and hemoglobin improved the predictive model for major adverse cardiovascular outcome (MACE; all-cause mortality or first heart failure hospitalization). They hypothesized this reflected diffuse myocardial fibrosis.

**Diagnostic and prognostic utility of cardiovascular magnetic resonance imaging in heart failure with preserved ejection fraction - implications for clinical trials**.

HFpEF is poorly characterized the potential insight from CMR after conventional imaging is not well defined. In this study, Kanagala et al. [20] studied 154 HFpEF patients. Patients underwent both transthoracic echocardiography (TTE) and CMR. CMR detected previously unknown pathology in 27% of patients, most commonly unsuspected coronary artery disease (many with silent myocardial infarction(MI), microvascular dysfunction, hypertrophic cardiomyopathy, and constriction.

**The interplay between metabolic alterations, diastolic strain rate and exercise capacity in mild heart failure with preserved ejection fraction: a cardiovascular magnetic resonance study**.

Heart failure is charazcterized by altered myocardial substrate metabolism. In this CMR spectroscopy study of 27 patients with HFpEF, Mahmod et al. [21] found HFpEF was associated with increased myocardial triglyceride content and reduced phosphocreatinine to adenosine triphosphate (PCr/ATP). On multivariate analysis, myocardial triglyceride content was independently associated with distaolic strain rate.

**Cardiac work is related to creatine kinase energy supply in human heart failure: a cardiovascular magnetic resonance spectroscopy study**.

It has been hypothesized that the supply of chemical energy may be insufficient in heart failure. In this study, Gabr and colleagues [22] studied 27 patients with mild-moderate heart failure with CMR spectroscopy. They found that heart failure was associated with reduced creatine kinase flux, average and peak cardiac mechanical work-rates, as well as cardiac mechanical efficiency.

**Myocardial native T2 measurement to differentiate light-chain and transthyretin cardiac amyloidosis and assess prognosis**.

The role of CMR in cardiac amyloidosis diagnosis and management is now well recognized. In this study, Ridouani et al. [23] studied 44 patients with light chain (AL) or transthyretin (ATTR) amyloidosis. The vast majority of subjects (82%) demonstrated late gadolinium enhancement (LGE). They found myocardial native T2 was significantly increased in AL in comparison with ATTR patients, but that native T2 did not impact survival. ECV was the best predictor of outcome.

**Non-invasive differentiation of idiopathic inflammatory myopathy with cardiac involvement from acute viral myocarditis using cardiovascular magnetic resonance imaging T1 and T2 mapping**.

Idiopathic inflammatory myopathy is a group of autoimmune diseases with systemic myositis that may involve the myocardium. In this retrospective study, Huber and colleagues [24] identified 20 subjects with idiopathic inflammatory myopathy and 20 with acute viral myocarditis. The best descriminator between acute viral myocarditis and idiopathic inflammatory myopathy was parametric mapping of skeletal muscle. Myocardial mapping did not discriminate between the two pathologies.

**Cardiovascular magnetic resonance in heart transplant patients: diagnostic value of quantitative tissue markers: T2 mapping and extracellular volume fraction, for acute rejection diagnosis**.

The clinical role of CMR in the heart transplant population with regards to rejection detection remains to be more fully defined. In this prospective study, Vermes and co-workers [25] studied 20 heart transplant patients undergoing 31 endomyocardial biopsies, including 7 who had acute rejection. Patients with actuer rejection had higher global T2 and basal ECV. They propose threshold T2 and ECV value for rejection.

**Effect of isolated left bundle-branch block on biventricular volumes and ejection fraction: a cardiovascular magnetic resonance assessment**.

The impact of abnormal septal contraction in isolated left bundle branch block (LBBB) on CMR metrics of biventricular function has not been fully defined. In this retrospective study, Akhtari et al. [26] identified 18 patients with isolated LBBB and found it to be associated with larger LV end-diastolic and end-systolic volumes and reduced LV ejection fraction (LVEF). There was no impact on right ventricular (RV) parameters.

## Coronary artery disease

Despite advances in diagnosis and management, coronary artery disease (CAD) remains a leading cause of morbidity and mortality. CMR continues to provide unique insights with regards to detection with vasodilator CMR stress testing [27–34], and the role of CMR in myocardial infarction detection [35], area at risk in acute ST elevation myocardial infarction (STEMI) [36–38] and post-infarction remodeling [39–42]. Despite its widespread clinical use for over a decade, improvements in LGE continued [43] in addition to advances in with dark blood [44, 45] and combined LGE and coronary artery imaging [46].

**Quantitative cardiovascular magnetic resonance perfusion imaging identifies reduced flow reserve in microvascular coronary artery disease**.

The exquisite sensitivity of CMR for assessing myocardial perfusion has raised interest intereste in its use for identifying microvascular disease. In this study, Zorach et al. [47] studied 44 patients with typical angina symptoms and risk factors for microvascular disease but no angiographic stenoses on coronary angiography. They found the cohort with microvascular disease risk factors had reduced myocardial perfusion reserve and stress myocardial perfusion even after adjusting for age, LV mass and gender. There were differences in native T1 or ECV between microvascular disease patients and healthy controls.

**Feasibility of cardiovascular magnetic resonance to detect oxygenation deficits in patients with multi-vessel coronary artery disease triggered by breathing maneuvers**.

Non-gadolinium based methods for detection of myocardial ischemia have continued to receive attention due to issues of concomitant renal dysfunction in the CAD population and concerns regarding long-term retention of gadolinium in healthy subjects. In this study, Fischer and co-workers [48] examine the role of breathing maneuvers to induce changes in myocardial oxygenation. Twenty-six patients with angiographic CAD underwent contrast free CMR at rest and during 60 s of paced hyperventilation followed by a prolonged breath-hold to induce a vasoactive stimulus. In comparison with healthy subjects, the CAD cohort had a significantly attenuated global myocardial oxygenation response during both hyperventilation and apnea. The breath-hold maneuver also unmasked regional oxygenation differences interritories subtended by a stenotic artery.

**Relationship between CMR-derived parameters of ischemia/reperfusion injury and the timing of CMR after reperfused ST-segment elevation myocardial infarction**.

To investigate the influence of CMR timing after reperfusion on CMR parameters of ischemia and reperfusion injury, Masci et al. [49] studied 163 reperfusion STEMI patients with CMR during the index hospitalization. T2 values of infarct and remote regions increased with increasing time from reascularization to CMR study, though infarct T2 exceeded remote myocardium for all intervals. Thus the time interval was *not* felt to impact the determination of myocardial area at risk.

**Incidence and predictors of left ventricular thrombus by cardiovascular magnetic resonance in acute ST-segment elevation myocardial infarction treated by primary percutaneous coronary intervention: a meta-analysis**.

Echocardiography without and with echo contrast is most commonly used for assessment of post-infarction ventricular function and thrombus, CMR been shown to be superior in many situations. In this meta-analysis, Bulluck and co-workers [50] did a MEDLINE and EMBASE datasearch and identified 10 studies reporting the incidence of LV thrombus on CMR within 1 month of STEMI. Overall, the incidence of LV thrombus was 6.3% with 96% of thrombi occurring with anterior STEMI. When only anterior STEMI and LVEF < 50% were considered, the incidence of LV thrombus was very high/19.2%! Compared with CMR, the sensitivity of transthoracic echocardiography (TTE) was only 29%, increasing to 70% with anterior STEMI and depressed LVEF.

**Dynamic changes in injured myocardium, very early after acute myocardial infarction, quantified using T1 mapping cardiovascular magnetic resonance**.

A bimodal pattern of myocardial edema has been suggested after acute MI. In this study, Alkhalil and colleagues [51] studied 31 patients who underwent 3 T CMR < 3 h, 24 h and 6 days after acute MI. Native T1 in the area at risk was reduced at 24 h and subsequently increased at 6 days. The extent of the area at risk by native T1 did not change between 3 h and 24 h, but did increase at 6 days.

**Quantitative cardiovascular magnetic resonance: extracellular volume, native T1 and 18F-FDG PET/CMR imaging in patients after revascularized myocardial infarction and association with markers of myocardial damage and systemic inflammation**.

Characterization of tissue integrity and inflammation after acute MI is predictive of patient outcomes. In this study, Kunze and co-workers [52] identified 25 patients without microvascular obstruction for 3 T CMR and positron emission tomography (PET) 5 days after MI. They found high intrapatient correlations of relative ECV, native T1, and ^18^F-FDG-PET. They conclude that absolute native T1 at the infarct core early after acute MI can be linked to systemic inflammation.

**Additive value of 3 T cardiovascular magnetic resonance coronary angiography for detecting coronary artery disease**.

Advances in coronary artery CMR continue with a focus on the integration of coronary CMR into the comprehensive CMR exam. In this study, Zhang and colleagues [53] performed contrast enhanced coronary artery CMR, stress CMR, and LGE at 3 T in 51 patients with suspected CAD. Coronary artery CMR success was achieved in over 90% of subjects. The inclusion of coronary artery CMR improved the overall sensitivity and diagnostic accuracy as compared with stress CMR and LGE alone.

## Outcomes/prognosis

Health care costs continue to rise faster than inflation with ever-expanding armamentarium of expensive therapies and monitoring options. As a result, there continues to be a need to focus on cost-effective medicine with avoidance of repetitive and redundant testing. CMR has often been recognized as a cost-effective/comprehensive test for evaluation and prognosis in myocarditis with T2 mapping [54], pulmonary vascular disease [55], implantable cardiodefibrillators (ICD) [56], and amyloidosis [57]. 2018 publications in this area included:

**The prognostic value of T1 mapping and late gadolinium enhancement cardiovascular magnetic resonance imaging in patients with light chain amyloidosis**.

Lin et al. studied 82 patients with AL amyloid and found AL patients had increased native T1 and ECV. During 8 months of follow-up, there was 25% mortality with ECV ≥44% and global LGE (but not native T1) found to be independent predictors of mortality [58].

**Asymptomatic myocardial ischemia forecasts adverse events in cardiovascular magnetic resonance dobutamine stress testing of high-risk middle-aged and elderly individuals**.

Current guidelines for assessing risk for MI discourage stress testing in asymptomatic subjects. In this prospective study, Stacey and co-workers [59] performed dobutamine stress CMR in 327 high risk patients without known CAD. Ischemia on dobutamine stress CMR identified asymptomatic patients at increased risk for future cardiovascular events/survival, especially in men.

**Prognostic value of myocardial strain and late gadolinium enhancement on cardiovascular magnetic resonance imaging in patients with idiopathic dilated cardiomyopathy with moderate to severely reduced ejection fraction**.

Both LV strain and LGE have prognostic valve in patients with heart failure. Pi et al. prospectively studied 172 patients with heart failure and reduced ejection fraction (HFrEF). During a median follow-up of 47 months, the presence of LGE was associated with MACE (heart transplantation, all cause death, heart failure hospitalization) [60].

## Population screening

Over the past 2 decades, CMR has emerged from research curiosity to mainstream including several large population screening studies including the Framingham Heart Study, Multi-Ethnic Study of Atherosclerosis (MESA) and UK Biobank, providing a broad window into the diversity of healthy populations and changes with aging and between genders and races in native T1 [61, 62], cardiac structure and function [63, 64].

**Diabetes mellitus and insulin resistance associate with left ventricular shape and torsion by cardiovascular magnetic resonance imaging in asymptomatic individuals from the multi-ethnic study of atherosclerosis**.

In this study from the MESA cohort, Yoneyama et al. [65] examined LV shape and longitudinal myocardial shortening and torsion. They found glucose metabolism disorders were associated with LV concentric remodeling, less spherical shape and reduced systolic shortening.

**Systemic arteriosclerosis is associated with left ventricular remodeling but not atherosclerosis: a TASCFORCE study**.

Arteriosclerosis is associated with future cardiovascular events. In this study, Weir-McCall and colleagues [66] reported on 1651 subjects free of clinical cardiovascular disease with 10 year- cardiovascular disease risk score of < 20%. Whole body CMR angiography demonstrated impaired total arterial compliance correlated with LV mass to volume ratio in both men and women.

**Determination of aortic stiffness using 4D flow cardiovascular magnetic resonance - a population-based study**.

Increased aortic stiffness is an independent predictor of cardiovascular disease. In this population-based study, Harloff and co-workers [67] prospectively studied 126 adults with 4D flow CMR at 3 T. They found that pulse wave velocity increased with age, was significantly lower in women, and increased with aortic diameter. After adjusting for age and gender, pulse wave velocity correlated with systolic, diastolic, and mean blood pressure.

**Cardiovascular magnetic resonance imaging in the prospective, population-based, Hamburg City Health cohort study: objectives and design**.

Several large population based CMR studies are now enrolling/following subjects. Among the most recently initiated is the Hamburg City Health cohort study. In this paper, Bohnen et al. [68] describe the proposed enrollment of 45,000 inhabitants Hamburg, Germany 45 to 74 years and plans for a relatively comprehensive CMR in 12,362. CMR measures will include conventional cine-CMR, native T1and T2 mapping, LGE, aortic/mitral valve flow, and aortic distensibility. Participants will be followed at 6 years for incident CAD, atrial fibrillation, and heart failure.

## Image analysis

A multitude of software analysis programs are now available to the CMR practitioner, each using somewhat proprietary methodology. CMR feature tracking and deep learning methods are receiving increasing attention.

**Comparison of left ventricular strains and torsion derived from feature tracking and DENSE CMR**.

CMR feature tracking is increasingly used to quantify cardiac mechanics. In this study, Wihner et al. [69] examined data from 88 patients who had displacement –encoding with stimulated echoes (DENSE) imaging. Data were compared with cine derived feature tracking analysis using TomTec Imaging Systems and Circle Cardiovascular Imaging software. Compared with DENSE, feature tracking overestimated the magnitude of basal and apical circumferential strain and dyssynchrony and underestimated torsion. Longitudinal strain had borderline acceptable agreement.

**Fully quantitative pixel-wise analysis of cardiovascular magnetic resonance perfusion improves discrimination of dark rim artifact from perfusion defects associated with epicardial coronary stenosis**.

Dark rim artifacts in first-pass CMR perfusion can mimic perfusion deficits and affect diagnostic accuracy for CAD detection. In this study of 76 patients undergoing regadenason stress CMR at 1.5 T, Ta and colleagues [70] found non-CAD subjects with dark rim artifacts had reduced myocardial blood flow in subendocardial than mid-myocardial and epicardial layers. Absolute stress myocardial blood flow differentiated CAD from non-CAD patients with high accuracy.

**Importance of operator training and rest perfusion on the diagnostic accuracy of stress perfusion cardiovascular magnetic resonance**.

Procedural success is associated with experience and training guidelines are often based on procedural volume to perform and maintain competency. The same may be for image interpretation. In this study, Villa and co-workers [71] evaluated the vasodilator stress CMR datasets of 53 patients with known or suspected CAD. Nine operators (3 each at Levels I, II, III CMR proficiency) blindly reviewed the case twice. Level 3 operators had a superior accuracy vs. Level 2 and Level 1 trained operators.

**A comparison of both DENSE and feature tracking techniques with tagging for the cardiovascular magnetic resonance assessment of myocardial strain**.

Myocardial strain is increasingly recognized as an important assessment for myocardial function and a precursor to depression of LVEF. In this study, Cao et al. [72] compared CMR tagging analyzed using harmonic phase (HARP) and DENSE images with three commercial software feature tracking programs in 87 subjects at 1.5 T. They found small but important differences in peak circumferential and longitudinal strain with large variations in radial strain among the vendor programs. Intra and inter-observer agreement were good for all except radial strain. They conclude that it is important to use the same technique and analytic software for clinical comparisons when monitoring patients for changes in strain.

**Automated cardiovascular magnetic resonance image analysis with fully convolutional networks**.

Automated image analysis with deep neural networks have shown great potential for image pattern recognition and segmentation with goals of faster, more accurate, and more reproducible image analysis. In this study, Bai and co-colleagues [1] examined data from 4875 subjects from the UK Biobank CMR dataset. They found the fully convolutional network to be highly accurate for segmenting the LV and RV from short-axis cine datasets as well as the left atrium and right atrium from long-axis datasets. There was minimal mean absolute differences with values similar to human experts.

**Fully automated, inline quantification of myocardial blood flow with cardiovascular magnetic resonance: repeatability of measurements in healthy subjects**.

Measurement of myocardial blood flow during vasodilator stress CMR, but there are issues with quantitative interpretation. In this study, Brown et al. [73] study 42 healthy subjects with rest and adenosine stress perfusion CMR using a fully automated, on-line myocardial perfusion tool. They found excellent intrastudy and interstudy measurements of global rest myocardial blood flow with good regional repeatability. Within subject coefficient of variation was also low.

## Congenital heart disease

The benefit of no ionizing radiation exposure and volumetric acquisitions continues to make CMR a preferred method for serial monitoring in patients with congenital heart disease (CHD) with 2017 publications demonstrating benefits in CHD [74–80].

**Cardiovascular magnetic resonance evidence of myocardial fibrosis and its clinical significance in adolescent and adult patients with Ebstein’s anomaly**.

CMR is well reconized for its superior ability to characterize ventricular volumes in CHD. In this consecutive series study by Yan and co-workers [81], 44 patients with unrepaired Ebstein’s anomaly were studied. LGE was found in nearly 25% of subjects, most commonly in the subendocardial septum. LV ECV was also elevated with increased ECV found to be independent of the presence of LGE. LGE and higher ECV were associated with larger functional RV volume and atrial RV volumes and worse New York Heart Association (NYHA) class.

**Impact of the cone operation on left ventricular size, function, and dyssynchrony in Ebstein anomaly: a cardiovascular magnetic resonance study**.

In addition to tricuspid regurgitation and RV dilation, patients with Ebstein’s anomaly are at risk for LV dysfunction. In this retrospective study of 20 Ebstein anomaly patients who underwent pre and post cone operation CMR, Beroukhim and colleagues [82] found that postoperatively, there was a decline in tricuspid regurgitant fraction, RV end-diastolic volume, and RV stroke volume, with unchanged RV ejection fraction. Conversely, theer was an increase in both LV end-diastolic vlume and LV stroke volume with no change in LVEF.

**Feasibility of 3D black-blood variable refocusing angle fast spin echo cardiovascular magnetic resonance for visualization of the whole heart and great vessels in congenital heart disease**.

Volumetric black-blood CMR has been hampered by long scan times and flow insensitivity. In this study, Henningsson and co-colleagues [83] implemented a 3D fast spin echo CMR method using sliced-selective excitation and non-selective refocusing pulses with variable flip angles to achieve constant echo signal for T1 andf T2 of vessel wall. They found that in healthy subjects and those with CHD, there was improved visualization of the superior pulmonary vein. In combination with 3D balanced steady state free precession (bSSFP) the 3D fast spin echo method substantially improved the success rate of cardiac morphological diagnosis.

**Impaired aortic distensibility and elevated central blood pressure in Turner Syndrome: a cardiovascular magnetic resonance study**.

Women with Turner Syndrome are at increased risk for aortic dissection. Wen et al. [84] explored arterial stiffness in 57 women with Turner Syndrome. They found Turner Syndrome was associated with descending aorta distensibility, primarily among those with aortic coarctation, with similar distensibility among Turner Syndrome patients without coarctation.

**Maldistribution of pulmonary blood flow in patients after the Fontan operation is associated with worse exercise capacity**.

Maldistribution of pulmonary artery blood flow is a potential complication of single ventricle palliation in the Fontan procedure. In this retrospective study, Alsaied and co-workers [85] studied 147 patients who underwent CMR and cardiopulmonary exercise testing within an average of 3 months. They found pulmonary artery blood flow maldistribution of to be common and associated with lower exercise capacity.

**Dynamic fetal cardiovascular magnetic resonance imaging using Doppler ultrasound gating**.

Dynamic fetal CMR may provide valuable adjunct to fetal echocardiography, but is difficult due to the lack of direct in-utero cardiac gating. In this study, Kording and colleagues [86] studied 15 fetuses at 1.5 T using a novel Doppler ultrasound device for CMR gating. They found that gating signals from the fetal heart were readily detected and provided for high-quality dynamic imaging of the fetal heart.

## Valvular heart disease/flow

Echocardiography continues to be the workhorse of clinical non-invasive cardiac imaging, but CMR is increasingly recognized as offering a unique insight, especially in situations in which echocardiographic acquisitions are poor or data are inconsistent. This is especially true for aortic stenosis [87–93] and mitral regurgitation [94, 95].

**Tricuspid flow and regurgitation in congenital heart disease and pulmonary hypertension: comparison of 4D flow cardiovascular magnetic resonance and echocardiography**.

Tricuspid regurgitation is a common complication of pulmonary artery hypertension and right sided congenital heart disease. In this prospective cross-sectional study, Driessen et al. [96] used CMR to assess tricuspid regurgitation using 4D-flow CMR and TTE. They found that 4D flow tricuspid valve effective flow correlated well with pulmonic valve 2D-flow CMR, and that 4D-flow CMR was reproducible for measurement of tricuspid valve flow and regurgitation.

**Differential flow improvements after valve replacements in bicuspid aortic valve disease: a cardiovascular magnetic resonance assessment**.

Abnormal aortic flow patterns in bicuspid valve disease may be partly responsible for the associated aortic dilation seen in these patients. To examine this, Bissell and co-workers [97] studied 90 subjects including 30 post-aortic valve replacement for bicuspid disease; 30 bicuspid patients, and 30 healthy controls. They found the majority of subjects with a mechanical aortic valve prosthesis or Ross procedure had normal aortic flow pattern, while all bioprosthetic prosthesis patients had abnormal flow patterns. They hypothesized that this differential in post-surgical flow may explain the different post-surgical aortic growth rates.

**Aortic flow patterns and wall shear stress maps by 4D-flow cardiovascular magnetic resonance in the assessment of aortic dilatation in bicuspid aortic valve disease**.

Patients with bicuspid aortic valve have ascending aortic dilation which may be caused by altered flow patterns and shear stress. In this study of 101 bicuspid aortic valve subjects, Rodriguez-Palomares and colleagues [98] found that different bicuspid aortic valve phenotypes have different flow patterns with anterior distribution with right-left leaflet fusion and posterior outflow jet in right-non coronary leaflet fusion. They hypothesized that the differential flow patterns may explain the different ascending aortic dilation morphotypes.

**Impact of surgical pulmonary valve replacement on ventricular strain and synchrony in patients with repaired tetralogy of Fallot: a cardiovascular magnetic resonance feature tracking study**.

Reparied tetralogy of Fallot with pulmonary valve replacement leads to changes in ventricular mechanics. In this prospective study, Balasubramanian et al. [99] underwent CMR before and 7 months after surgry. They found surgical pulmonary valve replacement led to improved LV global strain with no change in RV global strain. LV and RV synchrony patterns improved or were unchanged while interventricular synchrony worsened at the midventricular level.

**Multidimensional fetal flow imaging with cardiovascular magnetic resonance: a feasibility study**.

Flow CMR in the fetus is particularly challenging. In this study, Goolaub and colleagues [100] performed a novel phase-contrast CMR method in 5 pregnant women at 3 T. In healthy adults, they found their novel method agreed well with conventionally gated Cartesian acquisitions. In the pregnant cohort, fetal aortic flow was measured in the descending and ascending aorta and pulmonary artery.

The ability of CMR to acquire highly accurate cross sectional flow measurements in large and small arteries has long been recognized as a major attribute with increasing interest in 4D flow/shear assessments in patients with aortopathies. Our 2017 publications had a particular focus in this area [101–103]. 4D flow continued to be an area of intense interest.

**Left ventricular blood flow kinetic energy after myocardial infarction - insights from 4D flow cardiovascular magnetic resonance**.

MI leads to complex changes in LV morphology. In this study, Garg et al. [104] sought to examine LV blood flow kinetic energy in 48 patients after acute (*n* = 22) or chronic (*n* = 26) MI. They found LV kinetic energy to be reduced in MI patients with infarct size the most strongly associated with inplane kinetic energy.

**Test-retest variability of left ventricular 4D flow cardiovascular magnetic resonance measurements in healthy subjects**.

Quantification and visualization of LV blood flow is afforded by 4D CMR flow. To investigated this, Stoll and co-workers [105] examined test-retest variability in 45 healthy subjects. Patients underwent a second CMR study at the same visit or 1-2 months later. They found LV flow component’s volume and their associated kinetic energy values to be repeatable and stable over time. As might be expected, the variability over time was greater than the same visit measurements.

## Vascular imaging

CMR is more than “cardiac,” and *JCMR* welcomes submissions in the vascular arena. 2017 publications included studies imaging intravascular thrombus [106], vascular calcification [107], iron oxide vascular imaging [108], aortic imaging [109, 110], vascular wall imaging [111, 112] as well novel imaging methods such as quiescent-interval slice-selective (QISS) imaging [113].

**Atherosclerosis T1-weighted characterization (CATCH): evaluation of the accuracy for identifying intraplaque hemorrhage with histological validation in carotid and coronary artery specimens**.

Coronary high intensity plaques identified by CMR are associated with high-risk morphology lesions. In this pathology study, Liu et al. [114] studied 10 patients scheduled for carotid endarterectomy and 6 patients undergoing coronary artery endarterectomy with T1 weighted CMR. There was moderate agreement between in-vivo and ex-vivo imaging and plaque histology for intraplaque hemorrhage.

**Quantitative assessment of symptomatic intracranial atherosclerosis and lenticulostriate arteries in recent stroke patients using whole-brain high-resolution cardiovascular magnetic resonance imaging**.

Intracranial atherosclerotic stenoses have heterogeneous features. In this prospective study, Wang and co-workers [115] studied 29 symptomatic and 23 asymptomatic patients with intracranial atherosclerotic lesions with whole-brain high-resolution CMR. Symptomatic patients had larger enhancement plaque volume and a greater enhancement index. Regression analysis showed the enhancement index and average length of the lenticulostriate arteries were independent factors for predicting stroke.

**3D whole-brain vessel wall cardiovascular magnetic resonance imaging: a study on the reliability in the quantification of intracranial vessel dimensions**.

An important application of CMR vessel wall imaging is to monitor disease progression. To understand this further, knowledge of measurement reliability is needed. Zhang and colleagues [116] studied 34 healthy subjects and 10 patients with intracranial atherosclerosis using 3D and 2D intracranial vessel wall CMR. They found the excellent reproducibility and inter-method agreement for all morphologic measurements.

**Cardiovascular magnetic resonance black-blood thrombus imaging for the diagnosis of acute deep vein thrombosis at 1.5 Tesla**.

Deep vein thrombosis is a common clinical disorder. Chen et al. [117] studied the role of black-blood thrombus imaging with more conventional contrast-enhanced CMR venography in 30 acute deep venous thrombosis patients and 15 healthy subjects. In the acute phase, black-blood imaging demonstrated iso or hyperintense signal with similar diagnostic confidence as conventional contrast-enhanced imaging.

**Cardiovascular magnetic resonance imaging of aorto-iliac and ilio-femoral vascular calcifications using proton density-weighted in-phase stack of stars**.

Compared with CMR, computed tomography provides information regarding vessel calcification. To address this CMR deficiency, Serhal and co-workers [118] developed a proton density-weighted in-phase stack of stars CMR sequence. Patients were studied at 1.5 and 3 T. Qualitatively, the CMR stack of stars method showed good to excellent confidence in the detection of vascular calcifications with good-to-excellent inter-reader agreement. There was no overt performance difference between 1.5 and 3 T.

**Assessment of longitudinal distribution of subclinical atherosclerosis in femoral arteries by three-dimensional cardiovascular magnetic resonance vessel wall imaging**.

Lower extremity peripheral arterial disease represents a major health problem. In this study, Han and co-workers [119] studied 107 asymptomatic elderly (mean 72 years) subjects with 3D CMR femoral artery vessel wall imaging. They found femoral artery plaque in 65% with plaque most frequently in the popliteal artery and common femoral artery followed by proximal superficial femoral artery. The left femoral artery had a relatively smaller lumen area and a greater normalized wall index.

**Circulating levels of P-selectin and E-selectin relate to cardiovascular magnetic resonance-derid aortic characteristics in young adults from the general population, a cross-sectional study**.

It is uncertain whether endothelial cell adhesion molecules play a key role in early atherosclerosis. In this population based substudy of the Atherosclerosis-Monitoring and BIomarker-measurements in The YOUng (AMBITYON) cohort, Eikendal et al. [120] found that circulating P-selectin and E-selectin positively correlated with CMR-derived aortic wall thickness and aortic pulse wave velocity, suggesting these factors as a progenerator of early atherosclerosis.

## Atrial function and scar imaging

There has been increasing attention on the use of CMR to define the contribution of the atria in both health and disease [121–123].

**Validation of a rapid semi-automated method to assess left atrial longitudinal phasic strains on cine cardiovascular magnetic resonance imaging**.

Abnormal left atrial mechanical function is a marker for cardiac dysfunction. A fast method to measure left atrial strain is desireable. In this prospective study, Leng et al. [124] performed CMR in 30 patients with hypertrophic cardiomyopathy, 40 with HFrEF, 30 with heart failure and mid-range ejection fraction, 30 HFpEF, and 50 health controls. A fast long-axis strain measurement was peformed by automatically tracking the distance between the left atrioventricular junction and a user-defined point at the mid-posterior left atrial wall. There was good agreement between left atrial longitudinal strain and strain rate by conventional CMR and the fast assessment with significantly reduced left atrial strain and strain rate for all patient groups.

**The reproducibility of late gadolinium enhancement cardiovascular magnetic resonance imaging of post-ablation atrial scar: a cross-over study**.

CMR LGE methods have been used to identify post-pulmonary vein ablation scar, but the optimal timing post-gadolinium administration has not been explored. In this study, Chubb and colleagues [125] studied 40 patients after their first pulmonary vein ablation for atrial fibrillation with 1.5 T and 3 T electrocardiogram (ECG) and respiratory-navigator gated 3D LGE acquisition at 10, 20 and 30 min after contrast. Inter-scan reproducibility was good to excellent at 20 and 30 min, suggesting that a minimum of 20 min is preferred.

**Optimization of late gadolinium enhancement cardiovascular magnetic resonance imaging of post-ablation atrial scar: a cross-over study**.

In another study, Chubb and co-workers [126] reported on optomization of ECG and respiratory-navigator gated 3D LGE performed prior to and at twice at 3 months post-pulmonary vein. They found lower overall image quality at 3 T and at half-slice thickness. Contrast to noise and quality of scar delineation increased with time. They concluded that single dose contrast and a 20 to 30 min delay was optimal.

**Age-related changes of right atrial morphology and inflow pattern assessed using 4D flow cardiovascular magnetic resonance: results of a population-based study**.

To assess age-related changes of blood flow and geometry of the caval veins and right atrium, Wehrum et al. [127] studied an age-stratified sample of 126 subjects in the city of Freiburg, Germany. They found right atrial blood flow had a clockwise rotating helix without signs of turbulence in younger subjects with absent rotation in older subjects. There was also an age-relatd shift of the caval vein axis such that while the outlets of the superior and inferior vena caval veins were facing each other in the young, there was lateralization in the elderly. Mean and peak systolic blood flow also decreased with age.

## Interventional CMR

Interventional CMR offers the advantage of reduced radiation exposure for both the patient and the operator. This will likely be increasingly important with the ongoing growth of percutaneous structural heart disease options, with CMR investigators advancing the field in 2017 [128–132].

**Cardiovascular magnetic resonance guided ablation and intra-procedural visualization of evolving radiofrequency lesions in the left ventricle**.

One of the potential advantages of real time CMR is the opportunity to observe the impact of an intervention such as an ablation. In this swine study, Krahn et al. [133] performed a radiofrequency (RF) ablation in the LV using an actively-tracked CMR-enabled catheter. Edema at the ablation site was detected on T2 maps acquired as early as 3 min post-ablation and expanded throughout the 3 h observation period, while T1 derived lesions were relatively stable.

**Right heart catheterization using metallic guidewires and low SAR cardiovascular magnetic resonance fluoroscopy at 1.5 Tesla: first in human experience**.

CMR fluoroscopy allows for simultaneous assessment of cardiac function, flow and chamber pressure during cardiac catheterization. Commercial metallic gidewires were considered contraindicated during CMR due to concerns regarding radiofrequency induced heating. In this study, Campbell-Washburn and co-workers [134] used a low specific absorption rate imaging from gradient echo spiral acquisitions and a commercial nitinol guidewire for CMR fluoroscopy during right heart catheterization in 7 patients. They found neglible heating under all invitro conditions. In patients, chamber entry was successful in 100% of attempts vs. 94% without a guidewire. The time to reach each chamber was similar with and without a guidewire, but the guidewire was felt to impact useful catheter shaft conspicuity and enabled interactive modification of catheter shaft stiffness. Guideline tip visibility was poor.

## Technical innovation

CMR technical advances continue to lead to innovation in the use and application of CMR with 2017 reports involving myocardial perfusion [135, 136], native T1 [137], post-contrast T1 [138] and ECV [139], incoherent motion [140], vessel wall multiparametric mapping [141], and chemical exchange saturation transfer (CEST) [142]. There were also advances in 4D flow [143], high field 4D flow [144], real time flow [145], pulse wave velocity [146], left ventricular torsion [147] and CMR-based blood oximetry [148] in addition to first pass perfusion using hyperpolarized ^13^C urea [149], feature tracking [150, 151] and high throughput LV volumes [152].

**Comparison of fast multi-slice and standard segmented techniques for detection of late gadolinium enhancement in ischemic and non-ischemic cardiomyopathy - a prospective clinical cardiovascular magnetic resonance trial**.

In this study of patients with known or suspected LGE, Muehlberg and co-workers compared fast multi-slice and standard segmented techniqes for detection of LGE in 312 subjects with ischemic or non-ischemic cardiomyopathy including hypertrophic cardiomyopathy. They found fast multi-slice and standard segmented LGE sequences to be equivalent methods for the assessment of myocardial fibrosis [153].

**Gray blood late gadolinium enhancement cardiovascular magnetic resonance for improved detection of myocardial scar**.

Low scar-to-blood contrast in LGE imaging limits the visualization of subendocardial scars adjacent to the blood pool. Nulling the blood signal improves scar detection, but also reduced contrast between the myocardium and blood. In this study by Fahmy and colleagues [154], gray blood CMR was compared with black blood and conventional bright-blood LGE in phantom, swine and humans. Scar to blood contrast was improved with gray-blood LGE and gray-blood LGE detected more tissue scarring compared black blood and bright blood LGE.

**Regional assessment of carotid artery pulse wave velocity using compressed sensing accelerated high temporal resolution 2D CINE phase contrast cardiovascular magnetic resonance**.

CMR allows for assessment of aortic stiffness can be readily calculated, but assessment of smaller arteries has been more limited. In this technical study, Peper and co-workers [155] performed retrospective ECG gated 2D cine phase contrast CMR of the carotid artery using a compressed sensing accelerated high temporal resolution sequence at 1.5 T. They found their method provided similar flow curves as that with ultrasound at 1 ms temporal resolution, thereby enabling reliable pulse wave velocity estimates for transit times as short as 7.5 ms! Significant differences between carotid pulse wave velocity were found between young and elderly subjects.

**Simple motion correction strategy reduces respiratory-induced motion artifacts for k-t accelerated and compressed-sensing cardiovascular magnetic resonance perfusion imaging**.

CMR stress perfusion provides important prognostic information but current clinical sequences have limited spatial/temportal resolution and incomplete heart coverage. To address this, Zhou et al. [156] used respiratory motion compensation for k-t accelerated and compressed-sensing CMR perfusion to selectively correct for respiratory motion of the heart in a phantom and small patient study. They found their approach provided improved image quality.

**Cardiovascular cine imaging and flow evaluation using Fast Interrupted Steady-State (FISS) magnetic resonance**.

Existing cine imaging CMR sequences relay on bSSFP or spoiledgradient echo readouts, but have limitations due to rapid through-plane flow and off-resonance effects. Edeman and colleagues [157] examined a radial fast interrupted steady-state (FISS) method to overcome these limitations. They found cine FISS demonstrated a decrease in fat signal which imporved conspicuity of the coronary arteries while suppressing through-plane flow artifacts and improved fisualization of the aortic valve leaflets. Banding artifacts were also reduced.

**Noninvasive hematocrit assessment for cardiovascular magnetic resonance extracellular volume quantification using a point-of-care device and synthetic derivation**.

Calculation of ECV requires knowledge of the patient’s hematocrit. In this prospective study, Robison and co-workers [158] studied 123 subjects with laboratory hematocrit and a point-of-care device and by a synthetically derived hematocrit based on the relationship with blood pool T1 values. They found no significant differences between the point-of-care device and synthetic hematocrit and laboratory values at 1.5 T, but for discrimination of an abnormal ECV, the synthetic ECV approach had only moderate diagnostic performance.

**Importance of standardizing timing of hematocrit measurement when using cardiovascular magnetic resonance to calculate myocardial extracellular volume (ECV) based on pre- and post-contrast T1 mapping**.

ECV determination relies on knowledge of the patient’s hematocrit. In another prospective study study of 43 consecutive patients undergoing CMR, Engblom et al. [159] obtained venous blood samples upon patient arrival to the MR department and directly after the CMR examination with the patient remaining in the supine position. They found the hematocrit decreased after the CMR examination resulting in a significant change in the calculated ECV. They conclude that ECV variability could be reduced by standardizaing the timing of hematocrit measurement time relative to the CMR examination.

**Extracellular volume fraction measurements derived from the longitudinal relaxation of blood-based synthetic hematocrit may lead to clinical errors in 3 T cardiovascular magnetic resonance**.

If accurate, a synthetic hematocrit derived ECV would eliminate the need for a hematocrit test. In this 3 T study, Shang and co-workers [160] studied 226 subjects with hematocrit obtained on the day of the CMR with a synthetically derived hematocrit. They found that while the measured ECV strongly correlated with the synthetic ECV, the synthetic ECV would have incorrectly characterized 6-25% of patients.

**Two-center clinical validation and quantitative assessment of respiratory triggered retrospectively cardiac gated balanced-SSFP cine cardiovascular magnetic resonance imaging in adults**.

Sustained breath-holds remain a limitation for high spatial resolution cine bSSFP imaging. In this prospective two-center study, Pednekar et al. [161] compared breathhold bSSFP with a free breathing respiratory triggered bSSFP sequence with prospective cardiac arrhythmia rejection and retrospective cardiac gated reconstruction in real-time. They found global LV and RV functional parameters and LV mass from both sequences were in good agreement with similar blood-to-myocardial contrast. However, the combined clinical score for image quality was superior for the conventional breath-hold bSSFP approach.

**Real-time assessment of right and left ventricular volumes and function in children using high spatiotemporal resolution spiral bSSFP with compressed sensing**.

Real-time CMR cine for assessment of ventricular volumes and function during free breathing would be preferred in young children who often cannot cooperate with breathholding. Steeden and co-workers [162] studied 60 consecutive children with a novel real-time bSSFP spiral sequence with compressed sensing with conventional breath-hold bSSFP cine CMR. They found the real-time method had a much shorter acquisition time, but was associated with a small but significant overestimation of LV end-systolic volume and underestimation of end—diastolic volume, stroke volume, and ejection fraction.

**A multi-band double-inversion radial fast spin-echo technique for T2 cardiovascular magnetic resonance mapping of the heart**.

Double inversion recovery fast spin echo CMR sequences are typically used for black-blood T2-weighted imaging. However, these sequences suffer from slice inefficiency due to the non-selective inversion pulses. In this phantom and in-vivo study, Keerthivasan and colleagues [163] examined a multi-band encoded double inversion recovery radial fast spin-echo technique to simultaneously excite two slices. They found the relative contrast of the black-blood images were similar with the multi-band radial approach improving slice efficiency and allows for reconstruction of T2 maps for the excited slices.

## Animal models

Many technical advances are first implemented in the controlled environment of phantoms and ex-vivo/in-vivo animal models [35, 164–170].

**Quantification of myocardial infarct area based on TRAFFn relaxation time maps - comparison with cardiovascular magnetic resonance late gadolinium enhancement, T1rho and T2 in vivo**.

Early after MI, the infarct area consists primarily of necrotic tissue. In this mouse model of acute MI, Yla-Herttuala et al. [171] used CMR to characterize the infarct area and remote areas with rotating frame relaxation time mapping (T_RAFFn_) in addition to T_1p_ and T2. They found all relaxation time maps to have significant differences in relaxation time between the infarct and remote regions with increased signal intensities after gadolinium and areas with increased T_RAFFn_ in the MI area.

**Non-contrast assessment of microvascular integrity using arterial spin labeled cardiovascular magnetic resonance in a porcine model of acute myocardial infarction**.

Following acute MI, microvascular integrity and function may be compromised due to microvascular obstruction. In this swine study, Do and colleagues [172] performe arterial spin labeled CMR at rest and with vasodilator stress and after 90 min left anterior descending occlusion in a subset and repeat imaging. Regional myocardial blood flow increased with vasodilator stress. There was significant reduction in myocardial blood flow in the infarcted regional 1-2 days, 1-2 weeks and 4 weeks after MI. This was consistent with the perfusion deficit seen on first-pass CMR and with microvascular obstruction on LGE.

**Quantitative myocardial first-pass cardiovascular magnetic resonance perfusion imaging using hyperpolarized [1-(13)C] pyruvate**.

Absolute myocardial blood flow quantification would be preferred and hyperpolarized ^13^C pyruvate contrast offers this opportunity. In this theoretical and swine study by Fuetterer and co-workers [173], simulations and in-vivo swine images suggested that ^13^C pyruvate contrast provided sufficient SNR for both absolute and semi-quantitative perfusion.

**Simultaneous multi slice (SMS) balanced steady state free precession first-pass myocardial perfusion cardiovascular magnetic resonance with iterative reconstruction at 1.5 T**.

Simultaneous multi-slice perfusion imaging has the potential to acquire multiple slices, increasing myocardial coverage witout sacrificing in-plane spatial resolution. In this study, Nazir et al. [174] performed a simultaneous 6 slice and conventional 3 slice bSSFP sequence in 8 patients at rest. They found the simultaneous multi-slice had superior overall image quality and perceived signal-to-noise ratio with no difference in artifacts.

**Semi-automatic detection of myocardial trabeculation using cardiovascular magnetic resonance: correlation with histology and reproducibility in a mouse model of non-compaction**.

The definition of LV non-compaction remains ill-defined. In this genetic mouse model of non-compaction, Frandon et al. [175] found similar trabeculations at the basal, mid and apical LV levels, with strong correlation between the CMR and histologic trabeculated and compacted mass using semi-automated quantification software.

**Blood volume measurement using cardiovascular magnetic resonance and ferumoxytol: preclinical validation**.

A hallmark of heart failure is increased blood volume. Ramasawmy and colleagues [176] used ferumoxytol, a parenteral iron supplement with a long intravascular half-life and CMR to measure blood volume in swine. After adjustment for hematocrit, the derived blood volume agreed well with carbon monoxide measures, with good reproducibility.

## Miscellaneous topics

**Cardiovascular magnetic resonance assessment of acute cardiovascular effects of voluntary apnoea in elite divers**.

In this study, Eichhorn and colleagues [177] examined 17 elite apnea divers who performed 3 min breath-holds during real-time CMR. During prolonged apnea, peripheral oxygen saturation, LV ejection fraction, LV fractional shortening and heart rate all declined while blood flow was diverted to the cerebral regions.

**Artefacts in 1.5 Tesla and 3 Tesla cardiovascular magnetic resonance imaging in patients with leadless cardiac pacemakers**.

There are increasing data on the safety of transvenous pacing and CMR, but few data on leadless pacemakers. In this observational study, Kiblboeck and co-workers [178] performed CMR at least 6 weeks after Micra LCP leedless pacemaker implantation. The pacemaker was found to cause an arc-shaped artifact at the RV apex and 18% of LV segments were also markedly impacted. Artifacts were more frequent at 3 T as compared with 1.5 T. No clinical or device-related adverse events were found.

**Evaluation of skeletal muscle microvascular perfusion of lower extremities by cardiovascular magnetic resonance arterial spin labeling, blood oxygenation level-dependent, and intravoxel incoherent motion techniques**.

Lower extremity perfusion can be measured by multiple methods. In this study of healthy young and healthy and peripheral arterial disease elderly subjects, Suo et al. [179] performed arterial spin labeling (ASL), blood oxygenation level-dependent (BOLD) and intravoxel incoherent motion CMR after cuff-induced ischemia. No significant correlations were found among the three methods, but there was a significant correlation between T2* and ankle-brachial index in all muscle groups in peripheral arterial disease patients.

**An in-vivo comparison of stimulated-echo and motion compensated spin-echo sequences for 3 T diffusion tensor cardiovascular magnetic resonance at multiple cardiac phases**.

Though techinically challenging, diffuse tensor imaging (DTI) to examine myocardial microstructure has received increasing attention over the past few years. In this study, Scott and colleagues [180] compared breath-hold mid-ventricular short-axis stimulated echo (STEAM) and motion-compensate spin-echo DTI in 15 subjects. Successful acquisitions were greater with STEAM and there were significant differences in mean diffusivity, fractional anisotropy, tensor mode, transmural helical angle, and absolute second eigenvector angle. STEAM was therefore felt to be more reliable and values with different sequences should not be considered interchangeable.

**Diagnostic and prognostic significance of cardiovascular magnetic resonance native myocardial T1 mapping in patients with pulmonary hypertension**.

The impact of native T1 in pulmonary artery hypertension has not been examined. In this restrospective study, Saunders and colleagues [181] examined data from 490 consecutive patients undergoing 1.5 T CMR, including 369 with pulmonary artery hypertension. Patients with pulmonary artery hypertension had elevated native T1 in the septum at the RV insertion point. T1 did not contribute to predictive overall mortality.

**A comprehensive characterization of myocardial and vascular phenotype in pediatric chronic kidney disease using cardiovascular magnetic resonance imaging**.

Children with chronic kidney disease (CKD) have increased cardiovascular mortality. In this pediatric study of 120 children with mild, moderate and severe CKD, Cheang et al. [182] found CMR evidence for LV remodeling and reduced systolic and diastolic myocardial velocities in the CKD population. They hypothesized that novel CMR biomarkers may be useful in the early detection of abnormalities in this population.

**Cardiovascular magnetic resonance left ventricular strain in end-stage renal disease patients after kidney transplantation**.

Progressive CKD leading to end-stage renal disease is associated with significant cardiovascular morbidity and mortality. In this prospective study of 39 patients undergoing CMR prior to and 12 months after renal transplant, Gong and co-workers [183] found that renal transplant led to significant improvements in global circumferential strain and global radial strain but a decline in global longitudinal strain. The improvement in LV strain paralleled improvement in LVEF.

## Guidelines/reviews/position papers/SCMR endorsements

Guidelines continue to play an increasing role in the evaluation and patients with CMR increasingly recognized for providing unique metrics [184–188].

**Guidelines for training in cardiovascular magnetic resonance (CMR)**.

CMR training were first published by the SCMR in 2000 [189] and subsequently updated in 2007 [190]. In the 2018 update, Kim et al. [191] provide tracks for those able to obtain CMR training during cardiology fellowship or radiology/nuclear medicine residency as well as for physicians who have finished training and are in practice. The time and case load for Level I, II, III training are broadly similar to the 2007 publication.

**The growth and evolution of cardiovascular magnetic resonance: a 20-year history of the Society for Cardiovascular Magnetic Resonance (SCMR) annual scientific sessions**.

At the 20th annual SCMR scientific sessions, we had presentations by many of the SCMR founders. In this review, Lee and colleagues [192] summarize the history and tremendous growth of the SCMR through the lens of the founding members and annual scientific session program. Over 20 years, the annual scientific session attendance grew over 5 fold with a shift from early focus on CMR technique to clinical application and translation. They specifically identified the growth areas of CHD, clinical practice, and struction/function/flow.

**Journal of Cardiovascular Magnetic Resonance 2017**.

As with this publication, at the end of last year I summarized the 2017 publications in thematic constructs and provided a “State of our JCMR” perspective to our readership [6].

**Society for Cardiovascular Magnetic Resonance (SCMR) expert consensus for CMR imaging endpoints in clinical research: part I - analytical validation and clinical qualification**.

With the increasing acceptance of CMR as a preferred endpoint for clinical research due to high fidelity and reproducibility, Puntmann and colleagues [193] provided the first part of of an SCMR expert consensus statement for CMR imaging endpoints in clinical research detailing CMR’s analytic validation and clinical qualification.

**Aminophylline shortage and current recommendations for reversal of vasodilator stress: an ASNC information statement endorsed by SCMR**.

Both CMR and radionuclide myocardial perfusion share the use of vasodilators for providing differential perfusion to normal and ischemic territories. In mid-2018 there was a shortage of aminophylline, a pharmaceutical often used to reverse vasodilatory effects. This led to an American Society of Nuclear Cardiology (ASNC) informational statement led by Abidov et al. [194] regarding the use of aminophylline and alternative non-aminophylline based reversal protocols. Given the frequent use of vasodilator stress CMR, the SCMR endorsed the statement.

## Corrections

Minor corrections were made two Society publications and one manuscript:

Correction to: Clinical recommendations for cardiovascular magnetic resonance mapping of T1, T2, T2* and extracellular volume: A consensus statement by the Society for Cardiovascular Magnetic Resonance (SCMR) endorsed by the European Association for Cardiovascular Imaging (EACVI.

This manuscript was incorrectly listed as a review article and was changed to a position paper. The competing interests were also added [195]..

**Correction to: Diagnostic performance of semi-quantitative and quantitative stress CMR perfusion analysis: a meta-analysis**.

Corrections were made to Figs. 8-11 due to an error made in typesetting [196].

**Correction to: Simple motion correction strategy reduces respiratory-induced motion artifacts for k-t accelerated and compressed-sensing cardiovascular magnetic resonance perfusion imaging**.

Corrections were made to Fig. 1 of this original manuscript as one of the lines was not visible [197].

## Data Availability

Data sharing not applicable to this article as no datasets were generated or analyzed.

## Abbreviations

AL: Light chain
APC: Article processing charge
ASL: Arterial spin labeling
BOLD: Blood oxygenation level-dependent
bSSFP: Balanced steady state free precession
CAD: Coronary artery disease
CEST: Chemical exchange saturation transfer
CHD: Congenital heart disease
CKD: Chronic kidney disease
CME: Continuing medical education
DCM: Dilated cardiomyopathy
DENSE: Displacement encoding and stimulated echoes
DTI: Diffusion tensor imaging
ECG: Electrocardiogram
ECV: Extracellular volume fraction
HARP: Harmonic phase
HFpEF: Heart failure with preserved ejection fraction
HFrEF: Heart failure with reduced ejection fraction
ICD: Implanted cardiodefibrillator
ISMRM: International Society of Magnetic Resonance in Medicine
JCMR: *Journal of Cardiovascular Magnetic Resonance*
LBBB: Left bundle branch block
LGE: Late gadolinium enhancement
LV: Left ventricle/left ventricular
LVEF: Left ventricular ejection fraction
MACE: Major adverse cardiovascular event
MESA: Multi Ethnic Study of Atherosclerosis
MI: Myocardial infarction
MOLLI: Modified Look-Locker inversion recovery
NYHA: New York Heart Association
PET: Positron emission tomography
RV: Right ventricle/right ventricular
SCMR: Society for Cardiovascular Magnetic Resonance
STEAM: Stimulated echo
STEMI: ST-segment elevation myocardial infarction
T: Tesla
TTE: Transthoracic echo
